# Stability analysis of associative memory network composed of stochastic neurons and dynamic synapses

**DOI:** 10.3389/fncom.2013.00006

**Published:** 2013-02-21

**Authors:** Yuichi Katori, Yosuke Otsubo, Masato Okada, Kazuyuki Aihara

**Affiliations:** ^1^FIRST, Aihara Innovative Mathematical Modelling Project, Japan Science and Technology AgencyTokyo, Japan; ^2^Institute of Industrial Science, The University of TokyoTokyo, Japan; ^3^Graduate School of Frontier Science, The University of TokyoChiba, Japan; ^4^RIKEN Brain Science InstituteSaitama, Japan

**Keywords:** dynamic synapse, short-term plasticity, neural network, associative memory network, mean field model, bifurcation analysis

## Abstract

We investigate the dynamical properties of an associative memory network consisting of stochastic neurons and dynamic synapses that show short-term depression and facilitation. In the stochastic neuron model used in this study, the efficacy of the synaptic transmission changes according to the short-term depression or facilitation mechanism. We derive a macroscopic mean field model that captures the overall dynamical properties of the stochastic model. We analyze the stability and bifurcation structure of the mean field model, and show the dependence of the memory retrieval performance on the noise intensity and parameters that determine the properties of the dynamic synapses, i.e., time constants for depressing and facilitating processes. The associative memory network exhibits a variety of dynamical states, including the memory and pseudo-memory states, as well as oscillatory states among memory patterns. This study provides comprehensive insight into the dynamical properties of the associative memory network with dynamic synapses.

## 1. Introduction

Dynamic synapses change their transmission efficacy depending on the activity of the presynaptic neuron, and the postsynaptic response can be decreased (short-term depression) or increased (short-term facilitation) (Markram and Tsodyks, 1996; Tsodyks and Markram, 1997; Markram et al., 1998; Thomson, 2000; Wang et al., 2006). Synaptic transmission is carried out by the flow and diffusion of chemical components. Activation of a presynaptic neuron and generation of an action potential causes influx of calcium ions into the presynaptic membrane. A chemical reaction with the calcium ions triggers the release of the neurotransmitters and induces the post synaptic current. If many action potentials are generated in a short period of time, the calcium concentration and the fraction of releasable neurotransmitters change, and the transmission efficacy increases or decreases transiently. Change in the transmission efficacy is modeled by variables that represent the releasable neurotransmitters and the utilization parameter that defines the fraction of the neurotransmitter release by each action potential, reflecting the calcium concentration.

Stochastic neuron models with dynamic synapses and the corresponding mean field models have been proposed in previous studies, and their dynamical properties and possible roles of the dynamic synapses have been intensively investigated (Igarashi et al., 2010; Otsubo et al., 2010; Katori et al., 2012). Synaptic depression is known to enable neuronal gain control (Abbott et al., 1997), and to contribute to the destabilization of the network activity and generation of an oscillatory state (Pantic et al., 2002; Melamed et al., 2008; Otsubo et al., 2010). Synaptic facilitation is believed to enhance the working memory function in the prefrontal cortex (Mongillo et al., 2008). Furthermore, in a network with both depression and facilitation synapses, changes in the efficacy of dynamic synapses are suggested to reorganize the effective network structure, thereby contributing to flexible information processing in the prefrontal cortex (Katori et al., 2011).

An associative memory network retrieves a memory pattern according to their network dynamics in which the memory patterns are stored in their synaptic connections. Associative memory networks have also been well investigated (Anderson and Bower, 1972; Nakano, 1972; Amari, 1977; Hopfield, 1982; Adachi and Aihara, 1997). Dynamics of memory retrieval can be characterized as the convergence of the state of the network to a fixed-point attractor that corresponds to a stored memory pattern (Hopfield, 1982). In this type of conventional model of an associative memory network, the state of the network usually remains in the attractor. In contrast to this, in an associative memory network with the depression synapses, the memory retrieved state can be destabilized and the state of the network can move to another attractor that corresponds to another memory pattern. Such transitive dynamics among several memory patterns has been also investigated (Tsuda et al., 1987; Adachi and Aihara, 1997; Kanamaru et al., 2013). Although stochastic neural networks with depression and facilitation synapses have been studied (Torres et al., 2007; Mejias and Torres, 2009), a comprehensive understanding of the dynamics of associative memory networks with dynamic synapses has not yet been achieved.

In the present study, we focus on the associative memory network with stochastic neurons and dynamic synapses. In particular, we target stability analysis on the associative memory network with correlated memory patterns. The properties of the dynamic synapses are characterized by parameters that specify the time constants of recovery from an active state to a resting state of synapses. In the models of short-term plasticity the difference between depression and facilitation can be specified using theses parameters. We investigate how the dynamics of the associative memory network depends on these parameters.

In the following sections, first, we explain the model of a stochastic neural network with dynamic synapses. Next we derive the corresponding macroscopic mean field models that approximate the dynamical properties of the stochastic model. Furthermore, we analyze structural details of the dynamical system in the macroscopic mean field model, and we show how the network behavior and the memory-retrieval performance are influenced by noise intensity and the properties of the dynamic synapses. Finally, we discuss the results of the analyses from a viewpoint of neuroscience as well as possible future studies.

## 2. Materials and methods

### 2.1. Associative memory network with stochastic neurons and dynamic synapses

In this study, we use an associative memory network comprising *N* binary neurons. The state of the neuron is determined stochastically depending on inputs to the neuron. The state of the *i*th binary neuron at time *t* is denoted by the variable *s*_*i*_(*t*), which represents a resting state [*s*_*i*_(*t*) = 0] or an active state [*s*_*i*_(*t*) = 1] of the neuron. The state of the neuron changes according to the following probabilistic dynamics (Amit et al., 1985; Mejias and Torres, 2009):

(1) $$\text{Prob}[s_{i}(t+1)=1]=g_{\beta }(h_{i}(t)),$$

(2) $$\;g_{\beta }(h_{i}(t))=\frac{1}{2}\left(1+\tanh[\beta h_{i}(t)]\right),$$

where *g*_β_(*h*) is a neural response function with the noise intensity 1/β = *T*. The noise intensity *T* determines the smoothness of the response function; for *T* → +0 the model becomes deterministic. Note that we use {0, 1} to represent the neural activity in *s*_*i*_(*t*), whereas we use {−1, 1} to represent the memory patterns as we describe later. The equation

(3) $$h_{i}(t)=\sum_{j\;\neq \;i}^{N}J_{ij}[2s_{j}(t)x_{j}(t)u_{j}(t)/U_{\text{se}}-1]$$

represents the total input to the *i*th neuron. The quantity *J*_*ij*_ represents the absolute strength of the synaptic connection from the *j*th to *i*th neuron. *U*_se_ represents the fraction of released neurotransmitters in absence of depression and facilitation, and is the steady state value of the variable *u*_*i*_(*t*).

The properties of dynamic synapses activated by the *j*th neuron are modeled using the variables *x*_*j*_ and *u*_*j*_, which represent the fraction of releasable neurotransmitters and the utilization parameter, respectively (Tsodyks et al., 1998). The releasable neurotransmitters *x*_*j*_ decreases with activation of the synapse, which is triggered by the presynaptic neural activation. If there is no presynaptic activation, *x*_*j*_ recovers its steady state *x*_*j*_ = 1 with time constant τ_*R*_. The utilization parameter *u*_*j*_ increases with the activation of the synapse and recovers its steady state *u*_*j*_ = *U*_se_ with time constant τ_*F*_. This dynamics can be described by the following equations (Tsodyks and Markram, 1997; Tsodyks et al., 1998):

(4) $$x_{j}(t+1)=x_{j}(t)+\frac{1-x_{j}(t)}{\tau_{R}}-s_{j}(t)x_{j}(t)u_{j}(t),$$

(5) $$u_{j}(t+1)=u_{j}(t)+\frac{U_{\text{se}}-u_{j}(t)}{\tau_{F}}+U_{\text{se}}(1-u_{j}(t))s_{j}(t).$$

The efficacy of synaptic transmission is determined by the product of *x*_*j*_(*t*) and *u*_*j*_(*t*); the efficacy decreases (short-term depression) or increases (short-term facilitation) according to the parameters τ_*R*_, τ_*F*_, and *U*_se_.

Associative memory networks work well if the memory patterns are mutually orthogonal, but otherwise it does not necessarily work well. Moreover, in the associative memory network with depression synapses, the appearance of the oscillatory states is influenced by the similarity among the memory patterns (Otsubo et al., 2010). To evaluate the influence of the similarity among memory patterns in the network with both depression and facilitation synapses, we construct the associative memory network with correlated memory patterns by considering a parent memory pattern ξ and *p* child patterns ξ^μ^ (Amari, 1977; Toya et al., 2000) as follows:

(6) $$\xi =(\xi_{1},\ldots ,\xi_{N}),$$

(7) $$\xi^{\mu }=(\xi_{1}^{\mu },\ldots ,\xi_{N}^{\mu }),\mu =1,\ldots ,p.$$

**Figure 1:**
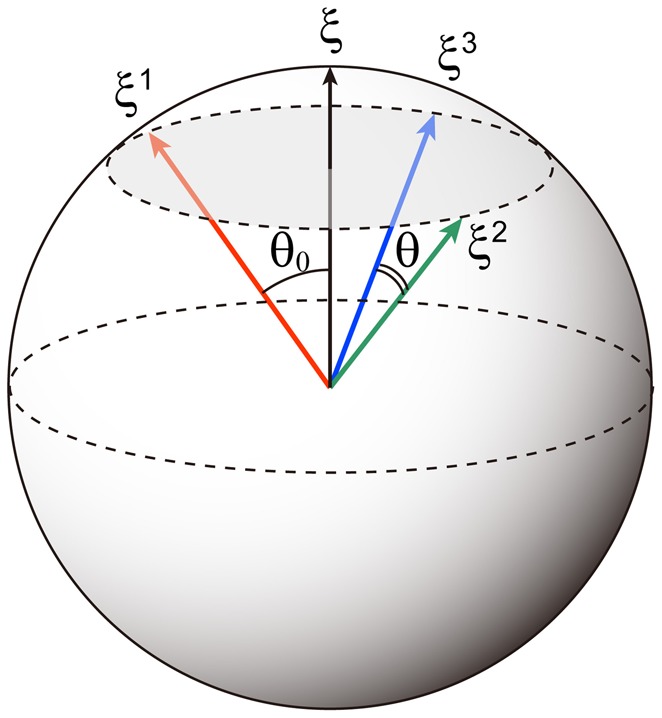
**Schematic of correlated memory patterns for *p* = 3.** The direction cosine between the parent pattern ξ and a memory pattern ξ^μ^ is cos θ_0_ = *b*. The direction cosine between two memory patterns is cos θ = *b*^2^.

Note that here we use the {−1, 1} to represents the memory patterns. A schematic of the relationship between these patterns for *p* = 3 is shown in Figure 1. Elements of the memory patterns are randomly generated according to the probability

(8) $$\text{Prob}[\xi_{i}=\pm 1]=\frac{1}{2},$$

(9) $$\text{Prob}[\xi_{i}^{\mu }=\pm 1]=\frac{1\pm b\xi_{i}}{2},$$

where *b* is the correlation level among memory patterns and takes values in the interval [0, 1]. For *b* = 0, child patterns are mutually orthogonal for *N* → ∞; for *b* = 1, the child patterns are the same as the parent pattern. Here we use the child patterns as the memory patterns. The direction cosine between memory patterns can described as $\cos\theta_{0}=\frac{1}{N}\sum_{i=1}^{N}\xi_{i}\xi_{i}^{\mu }=b$ and $\cos\theta =\frac{1}{N}\sum_{i=1}^{N}\xi_{i}^{\mu }\xi_{i}^{\nu }=b^{2}$, where μ ≠ ν (Otsubo et al., 2010).

According to the Hebbian rule, we use the following absolute strength of synaptic connection *J*_*ij*_:

(10) $$J_{ij}=\frac{1}{N}\sum_{\mu }^{p}\xi_{i}^{\mu }\xi_{j}^{\mu },$$

where the self-recurrent connection does not exist (i.e., *J*_*ii*_ = 0). The absolute strength represents the synaptic response on the connected neurons when the synapses do not undergo any depression and facilitation. The connections with positive or negative values of the absolute strength correspond to excitatory or inhibitory synaptic connections, respectively.

### 2.2. Mean field theory

To analyze the macroscopic properties of the associative memory network with stochastic neurons, we consider dynamical mean field theory with the sublattice method (Coolen, 2001; Otsubo et al., 2010), which allows us to analyze the mean field model with the non-homogeneous network structure of the associative memory network.

First, we derive the microscopic mean field model by taking the noise average of each variable in the stochastic neural network model. We get the following equations from Equations (1) to (3):

(11) $$\langle s_{i}(t+1)\rangle =g_{\beta }(\langle h_{i}(t)\rangle ),$$

(12) $$\langle h_{i}(t)\rangle =\sum_{j\;\neq \;i}^{N}J_{ij}[2\langle s_{j}(t)x_{j}(t)u_{j}(j)\rangle /U_{\text{se}}-1].$$

Because of the non-convexity of the response function *g*_β_ and the excitatory feedback connection, the network can stabilize the self-sustained active states (Barbieri and Brunel, 2007). Similarly to Equation (11), we obtain the following equations corresponding to Equations (4) and (5):

(13) $$\langle x_{j}(t\text{​}+\text{​}1)\rangle \text{​}=\text{​}\langle x_{j}(t)\rangle \text{​}+\text{​}\frac{1-\langle x_{j}(t)\rangle }{\tau_{R}}-\langle s_{j}(t)x_{j}(t)u_{j}(t)\rangle ,$$

(14) $$\langle u_{j}(t\text{​}+\text{​}1)\rangle \text{​}=\text{​}\langle u_{j}(t)\rangle \text{​}+\text{​}\frac{U_{\text{se}}-\langle u_{j}(t)\rangle }{\tau_{F}}+U_{\text{se}}\langle (1-u_{j}(t))s_{j}(t)\rangle .$$

Here, we assume that the correlations among variables *s*_*j*_(*t*), *x*_*j*_(*t*), and *u*_*j*_(*t*) are negligible on the basis of the following considerations. The correlations among the variables *s*_*j*_(*t*), *x*_*j*_(*t*), and *u*_*j*_(*t*) can be separated into three pairs. The state of *s*_*j*_(*t*) is determined by the state of other neurons in the previous time step, and the state of *x*_*j*_(*t*) and *u*_*j*_(*t*) are determined by the state of each variable in the previous time step. Thus, the correlation between *s*_*j*_(*t*) and *x*_*j*_(*t*) is of the order 1/*N*, and this correlation disappears as *N* → ∞ (Igarashi et al., 2010). Similarly, the correlation between *s*_*j*_(*t*) and *u*_*j*_(*t*) also disappears as *N* → ∞. Accordingly, we assume the following independent relations between variables:

(15) $$\langle s_{j}(t)x_{j}(t)u_{j}(t)\rangle =\langle s_{j}(t)\rangle \langle x_{j}(t)\rangle \langle u_{j}(t)\rangle ,$$

(16) $$\langle s_{j}(t)u_{j}(t)\rangle =\langle s_{j}(t)\rangle \langle u_{j}(t)\rangle .$$

Note that the independency between *x*_*j*_(*t*) and *u*_*j*_(*t*) is reported to hold if there is no facilitation (Tsodyks et al., 1998). Thus, we evaluate the validity of this assumption by comparison between the simulation and the mean field model derived by this assumption. As we show in “Results” section, the mean field model shows good approximations. By using these relations (15) and (16), the microscopic mean field model is derived as

(17) $$m_{i}(t\text{​}+\text{​}1)\text{​}=\text{​}g_{\beta }\left[\sum_{j\;\neq \;i}^{N}J_{ij}\left(2m_{j}(t)X_{j}(t)U_{j}(t)/U_{\text{se}}-1\right)\right],$$

(18) $$X_{i}(t\text{​}+\text{​}1)\text{​}=\text{​}X_{i}(t)+\frac{1-X_{i}(t)}{\tau_{R}}-m_{i}(t)X_{i}(t)U_{i}(t),$$

(19) $$U_{i}(t\text{​}+\text{​}1)\text{​}=\text{​}U_{i}(t)+\frac{U_{\text{se}}-U_{i}(t)}{\tau_{F}}+U_{\text{se}}(1-U_{i}(t))m_{i}(t),\;$$

where *m*_*i*_(*t*) ≡ 〈*s*_*i*_(*t*)〉, *X*_*i*_(*t*) ≡ 〈*x*_*i*_(*t*)〉, and *U*_*i*_(*t*) ≡ 〈*u*_*i*_(*t*)〉.

We now derive the mean field model that describes the macroscopic dynamical properties of the associative memory network. Here we use the sublattice method (Coolen, 2001) with *p*-dimensional pattern vectors η = (η^1^, …, η^*p*^)^*T*^ ∈ {−1, 1}^*p*^. A set of neurons {1, …, *N*} is divided into 2^*p*^ groups on the basis of these pattern vectors. Suppose that ${\bar{\xi }}_{i}={\left(\xi_{i}^{1},\ldots ,\xi_{i}^{p}\right)}^{T}\in {\left\{-1,1\right\}}^{p}$, a sublattice is defined as a set of neurons belonging to a given pattern vector. The sublattice belonging to the pattern vector η is defined as

(20) $${ℐ}_{\eta }=\{i|{\bar{\xi }}_{i}=\eta \},$$

(21) $$\{1,\ldots ,N\}=\underset{\eta }{\cup }{ℐ}_{\eta },$$

where ℐ_η_ is called a sublattice.

The absolute strength of synaptic connection (Equation 10) can be rewritten with the expression of the sublattice as follows:

(22) $$\begin{array}{l} J_{ij}=\frac{1}{N}\sum_{\mu =1}^{p}\eta^{\mu }\eta^{\prime \mu }=\frac{1}{N}\eta \cdot \eta^{\prime },\\ \text{ for }i\in {ℐ}_{\eta },\text{ and }j\in {ℐ}_{\eta^{\prime }}. \end{array}$$

We assumed that neurons within the same sublattice ℐ_η_ follow the same dynamics and that the variables in the microscopic mean field model (Equations 17–19) can be described as

(23) $$m_{i}(t)=m_{\eta }(t),X_{i}(t)=X_{\eta },\text{ and }U_{i}(t)=U_{\eta }\text{ for }i\in {ℐ}_{\eta }.$$

With these assumptions, we obtain the following macroscopic mean field model of the associative memory network:

(24) $$m_{\eta }(t+1)=F_{m_{\eta }}(\{m_{\eta }(t)\},\{X_{\eta }(t)\},\{U_{\eta }(t)\}),$$

(25) $$X_{\eta }(t+1)=F_{X_{\eta }}(\{m_{\eta }(t)\},\{X_{\eta }(t)\},\{U_{\eta }(t)\}),$$

(26) $$U_{\eta }(t+1)=F_{U_{\eta }}(\{m_{\eta }(t)\},\{X_{\eta }(t)\},\{U_{\eta }(t)\}),$$

where

(27) $$\begin{array}{l} F_{m_{\eta }}(\{m_{\eta }(t)\},\{X_{\eta }(t)\},\{U_{\eta }(t)\})\\ \;=g_{\beta }\left(\text{​}\sum_{\eta^{\prime }}p_{\eta^{\prime }}\eta \cdot \eta^{\prime }\left(2m_{\eta^{\prime }}(t)X_{\eta^{\prime }}(t)U_{\eta^{\prime }}(t)/U_{\text{se}}-1\right)\text{​}\right)\text{​}, \end{array}$$

(28) $$\begin{array}{l} F_{X_{\eta }}(\{m_{\eta }(t)\},\{X_{\eta }(t)\},\{U_{\eta }(t)\})\\ \;=X_{\eta }(t)+\frac{1-X_{\eta }(t)}{\tau_{R}}-m_{\eta }(t)X_{\eta }(t)U_{\eta }(t), \end{array}$$

(29) $$\begin{array}{l} F_{U_{\eta }}(\{m_{\eta }(t)\},\{X_{\eta }(t)\},\{U_{\eta }(t)\})\\ \;=U_{\eta }(t)+\frac{U_{\text{se}}-U_{\eta }(t)}{\tau_{F}}+U_{\text{se}}(1-U_{\eta }(t))m_{\eta }(t), \end{array}$$

where *p*_η_ = |ℐ_η_|/*N* denotes the relative sublattice size.

We represent the steady state for the macroscopic mean field model by ${\bar{m}}_{\eta }$, ${\bar{X}}_{\eta }$, and ${\bar{U}}_{\eta }$. The steady state for the Equations (24–26) with (*t* → ∞) is given by the following self-consistent equations:

(30) $${\bar{m}}_{\eta }=g_{\beta }\left(\sum_{\eta^{\prime }}P_{\eta^{\prime }}\eta .\eta^{\prime }\left(\frac{2{\bar{m}}_{\eta^{\prime }}(1+\tau_{F}{\bar{m}}_{\eta^{\prime }})}{\begin{array}{l} 1+(\tau_{F}+\tau_{R})U_{\text{se}}{\bar{m}}_{\eta^{\prime }}\\ \;+U_{\text{se}}\tau_{F}\tau_{R}{\bar{m}}_{\eta^{\prime }}^{2} \end{array}}-1\right)\right),$$

(31) $${\bar{X}}_{\eta }=\frac{1}{1+\tau_{R}{\bar{U}}_{\eta }{\bar{m}}_{\eta }},$$

(32) $${\bar{U}}_{\eta }=\frac{U_{\text{se}}(1+\tau_{F}{\bar{m}}_{\eta })}{1+\tau_{F}U_{\text{se}}{\bar{m}}_{\eta }}.$$

To investigate the stability of the system given by Equations (24–26) around the steady state given by Equations (30–32), we consider the locally linearized equations with small perturbations δ*m*_η_(*t*), δ*X*_η_(*t*), and δ*U*_η_(*t*) around the steady state as follows:

(33) $$m_{\eta }(t)={\bar{m}}_{\eta }+\delta m_{\eta }(t),$$

(34) $$X_{\eta }(t)={\bar{X}}_{\eta }+\delta X_{\eta }(t),$$

(35) $$U_{\eta }(t)={\bar{U}}_{\eta }+\delta U_{\eta }(t).$$

We obtain the following locally linearized equations on the small perturbations around the steady state with Jacobian matrix *K*.

(36) $$\left(\begin{array}{c} \delta m_{\eta }(t+1)\\ \delta X_{\eta }(t+1)\\ \delta U_{\eta }(t+1) \end{array}\right)=K\left(\begin{array}{c} \delta m_{\eta }(t)\\ \delta X_{\eta }(t)\\ \delta U_{\eta }(t) \end{array}\right).$$

The stability of the system can be determined by the eigenvalues of the Jacobian matrix on the steady state; the stability is distinguished by the absolute value of the eigenvalues. Elements on the Jacobian matrix *K* are given as

(37) $$\frac{\partial F_{m_{\eta }}}{\partial m_{\eta^{\prime }}}=g_{\beta }^{\prime }(h)p_{\eta^{\prime }}\eta \cdot \eta^{\prime }(2X_{\eta^{\prime }}(t)U_{\eta^{\prime }}(t)/U_{\text{se}}),$$

(38) $$\frac{\partial F_{m_{\eta }}}{\partial X_{\eta^{\prime }}}=g_{\beta }^{\prime }(h)p_{\eta^{\prime }}\eta \cdot \eta^{\prime }(2m_{\eta^{\prime }}(t)U_{\eta^{\prime }}(t)/U_{\text{se}}),$$

(39) $$\frac{\partial F_{U_{\eta }}}{\partial U_{\eta^{\prime }}}=g_{\beta }^{\prime }(h)p_{\eta^{\prime }}\eta \cdot \eta^{\prime }(2m_{\eta^{\prime }}(t)X_{\eta^{\prime }}(t)/U_{\text{se}}),$$

where

(40) $$g_{\beta }^{\prime }(h)=\frac{\beta }{2}\left(1-\tanh^{2}(\beta h)\right),$$

(41) $$h=\sum_{\eta^{\prime }}p_{\eta^{\prime }}\;\eta \cdot \eta^{\prime }\left(2m_{\eta^{\prime }}X_{\eta^{\prime }}U_{\eta^{\prime }}/U_{\text{se}}-1\right).$$

Furthermore, the remaining matrix elements are given by

(42) $$\frac{\partial F_{X_{\eta }}}{\partial m_{\eta^{\prime }}}=-U_{\eta }X_{\eta }\delta_{\eta ,\eta^{\prime }},$$

(43) $$\frac{\partial F_{X_{\eta }}}{\partial X_{\eta^{\prime }}}=\left(\left(1-\frac{1}{\tau_{R}}\right)-m_{\eta }U_{\eta }\right)\delta_{\eta ,\eta^{\prime }},$$

(44) $$\frac{\partial F_{X_{\eta }}}{\partial U_{\eta^{\prime }}}=-m_{\eta }X_{\eta }\delta_{\eta ,\eta^{\prime }},$$

(45) $$\frac{\partial F_{U_{\eta }}}{\partial m_{\eta^{\prime }}}=U_{\text{se}}(1-U_{\eta })\delta_{\eta ,\eta^{\prime }},$$

(46) $$\frac{\partial F_{U_{\eta }}}{\partial X_{\eta^{\prime }}}=0,$$

(47) $$\frac{\partial F_{U_{\eta }}}{\partial U_{\eta^{\prime }}}=\left(\left(1-\frac{1}{\tau_{F}}\right)-U_{\text{se}}m_{\eta }\right)\delta_{\eta ,\eta^{\prime }},$$

where δ_η, η′_ is Kronecker's delta, namely, δ_η, η′_ is 1 if the η = η′, and 0 otherwise. By using this Jacobian matrix, we analyze the stability of the steady states in the following section.

In the following analysis, we fix the number of stored pattern to be *p* = 3. In this case, neurons can be divided into eight sublattices with the following combination of η:

(48) $$\begin{array}{l} \text{​​​​​​​​​​​​​​}\eta \in \{{\left(1,\text{ ​}1,\text{ ​}1\right)}^{T}\text{​},\;{\left(1,\text{ ​}1,\;-1\right)}^{T}\text{​},\;{\left(1,\;-1,\text{ ​}1\right)}^{T}\text{​},\;{\left(1,\;-1,\;-1\right)}^{T},\text{​}\\ \text{ ​​​​​​}{\left(-1,\text{ ​}1,\text{ ​}1\right)}^{T}\text{​},\;{\left(-1,\text{ ​}1,\;-1\right)}^{T}\text{​},\;{\left(-1,\;-1,\text{ ​}1\right)}^{T}\text{​},\;{\left(-1,\;-1,\;-1\right)}^{T}\}.\; \end{array}$$

Since the memory patterns are provided by Equations (8) and (9), the number of neurons |ℐ_η_| in the sublattice ℐ_η_ is given as follows (Otsubo et al., 2010):

(49) $$\begin{array}{l} |{ℐ}_{\eta }|\text{​ }=\text{ ​}\left\{ \begin{array}{ll} N(1+3b^{2})/8, & \text{if }\eta ={\left(1,\;1,\;1\right)}^{T},\;{\left(-1,\;-1,\;-1\right)}^{T}\text{​},\\ N(1-b^{2})/8, & \text{otherwise}. \end{array}\right. \end{array}$$

The model with *p* = 3 is composed of 24 variables in total.

## 3. Results

In this section, we present the results of simulation in the stochastic model and of analyses of the macroscopic behavior in the associative memory model with dynamic synapses. In particular, we analyze the changes in the structure of the dynamics, depending on the parameters *T*, τ_*F*_, τ_*R*_, and *U*_se_.

To quantify the similarity between the state of the network *s*(*t*) and the μth memory pattern ξ^μ^, we use an overlap given by

(50) $$M^{\mu }(t)=\frac{1}{N}\sum_{i\;=\;1}^{N}\;\xi_{i}^{\mu }[2s_{i}(t)-1].$$

In the Equation (50), if 2*s*_*i*_(*t*) − 1 is equal to ξ^μ^_*i*_, ∀*i*, then *M*^μ^(*t*) = 1. This means that if the state of neurons completely matches the μth memory pattern, the overlap becomes unity. In the formulation of the macroscopic mean field model, the above equation can be rewritten as follows:

(51) $$M^{\mu }(t)=\sum_{\eta^{\prime }}p_{\eta^{\prime }}{\eta^{\prime }}^{\;\mu }[2m_{\eta^{\prime }}(t)-1].$$

Furthermore, the state of the network is classified according to the symmetry of the overlaps by using the effective dimension (ED) which is defined in the following. We consider only the case with *p* = 3. If the values of three overlaps at time *t* are equal or nearly equal, namely, if they satisfy |*M*^μ^(*t*) − *M*^ν^(*t*)| < ϵ, ∀(μ, ν) ∈ {(1, 2), (2, 3), (3, 1)}, then ED(*t*) = 1, where ϵ = 10^−5^. If the values of all the overlaps are different i.e., if they satisfy |*M*^μ^(*t*) − *M*^ν^(*t*)| > ϵ, ∀(μ, ν) ∈ {(1, 2), (2, 3), (3, 1)}, then ED(*t*) = 3. Otherwise, namely, if the values of two of three overlaps are equivalent, ED(*t*) = 2. The mean effective dimension (MED) is defined as MED = ∑^*L*^_*t* = 1_ ED(*t*)/*L*, where *L* is the length of a given time course.

We classified the state of the network according to the overlaps and the ED. There are four different types of steady state (fixed point), described as follows. In the memory state (MEM), one of the memory patterns or inverted memory patterns is retrieved. In the symmetric (asymmetric) mixed state [SMIX(AMIX)], one of the symmetric (asymmetric) mixture of the memory patterns is retrieved. In the paramagnetic state (PARA), the network does not retrieve any patterns and the state of each neuron is random. The oscillatory states have been classified according to the ED of the macroscopic mean field model giving rise to three oscillatory regimes: OS1, OS2, and OS3 states, which satisfy MED = 1, 1 < MED ≤ 2, and 2 < MED ≤ 3, respectively.

**Figure 2:**
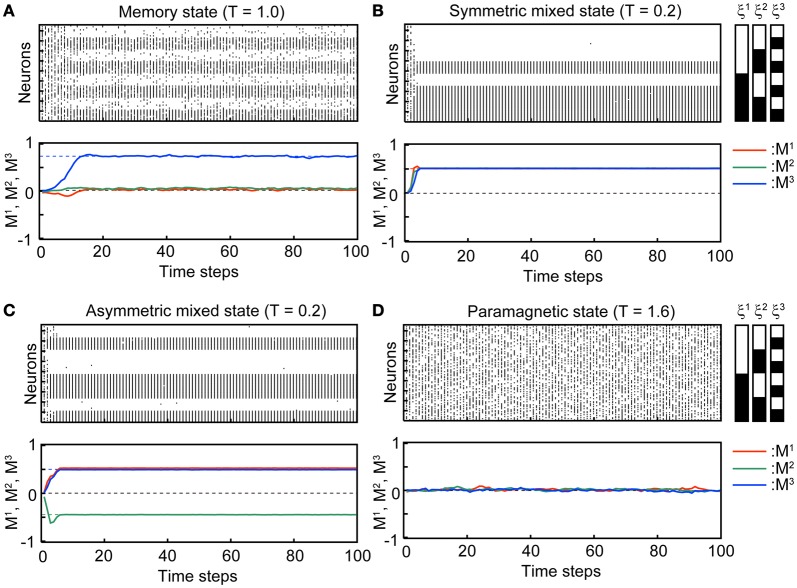
**Transient processes in the simulation where the associative network converges to the steady state with *U*_se_ = 0.1, *b* = 0.2, τ_*R*_ = 4, and τ_*F*_ = 2.** The initial states *s*_*i*_(0) of the simulation are set randomly so that the overlaps are almost zero in the initial state. **(A)** Memory state (*T* = 1.0). **(B)** Symmetric mixed state (*T* = 0.2). **(C)** Asymmetric mixed state (*T* = 0.2). **(D)** Paramagnetic state (*T* = 1.6). In the top panel of each subfigure, dots indicate the active state of the neurons [*s*_*i*_(*t*) = 1]. 96 of 10^4^ neurons are displayed. The indices of the neurons on the vertical axis are sorted according to the stored memory patterns. The black and white striped pattern indicates the stored memory pattern. Each black and white indicate 1 and 0, respectively. In the second panel of each subfigure, the overlaps between *M*^1^, *M*^2^, and *M*^3^ are indicated by red, green, and blue curves, respectively. The solid curves are the simulation in the stochastic model and dashed lines indicate the steady state in the macroscopic mean field model.

Figure 2 shows typical time courses indicating that the state of the network converges to the steady states. The top panels in each subfigure in Figure 2 show a raster plot; the dots indicate the active state of the neuron with *s*_*i*_(*t*) = 1. The initial states of the simulation in the stochastic model are *x*_*i*_(*t*) = 1, *u*_*i*_(*t*) = *U*_se_, and *s*_*i*_(*t*) are set to be 0 or 1 randomly so that the overlaps are almost zero in the initial state. We used *N* = 10^4^ neurons in the simulation. The bottom panels show overlaps *M*^1^(*t*), *M*^2^(*t*), and *M*^3^(*t*) of the stochastic model (solid curves) and its corresponding steady states in the macroscopic mean field model (dashed lines). Appearance of the steady states of the stochastic model is consistent with the corresponding macroscopic mean field model.

In the MEM state (Figure 2A), one of the memory patterns or inverted memory patterns is retrieved. The state of the network converges to a steady state, which corresponds to a stable fixed point in the macroscopic mean field model. The steady state can be represented with the overlaps as e.g., (*M*^1^, *M*^2^, *M*^3^) = (M, M^*^, M^*^), where *M* and M^*^ satisfy *M* > M^*^ > 0, and the corresponding memory pattern is ξ^1^. There are six possible MEM states: the states obtained by the permutations on the three memory patterns and its inversion. Figure 2A shows a typical time course of the process of convergence to the MEM state (to the memory pattern ξ^3^ in the Figure 2A ) in the stochastic model.

In the SMIX state (Figure 2B), the mixture of the memory patterns or the inverted memory patterns is retrieved. There are two possible SMIX states; the SMIX states are represented as (*M*^1^, *M*^2^, *M*^3^) = ($\bar{M}$, $\bar{M}$, $\bar{M}$) for the mixture of the stored patterns and (*M*^1^, *M*^2^, *M*^3^) = (−$\bar{M}$, −$\bar{M}$, −$\bar{M}$) for its inverse, where $\bar{M}$ > 0. The corresponding memory patterns are sgn(ξ^1^ + ξ^2^ + ξ^3^) and −sgn(ξ^1^ + ξ^2^ + ξ^3^), respectively. Figure 2B shows a typical time course that the network converges to the SMIX state [to the mixture of the stored patterns sgn(ξ^1^ + ξ^2^ + ξ^3^) in the Figure 2B].

In the AMIX state (Figure 2C), one of the asymmetric mixture of the memory patterns is retrieved. The AMIX state can be represented as e.g., (*M*^1^, *M*^2^, *M*^3^) = (−*M*″, *M*′, *M*′), where *M*′ > *M*″ > 0, and the corresponding memory pattern is sgn(−ξ^1^ + ξ^2^ + ξ^3^). There are six possible AMIX states: the states obtained by the permutations on the three memory patterns and its inversion. Figure 2C shows a typical time course of the state of the network when the state converges to the AMIX state that corresponds to the pattern sgn(ξ^1^ − ξ^2^ + ξ^3^).

In the PARA state, the state of each neuron is random. Thus, the PARA state is represented as (*M*^1^, *M*^2^, *M*^3^) = (0, 0, 0). Figure 2D shows that the network stays on the PARA state.

**Figure 3:**
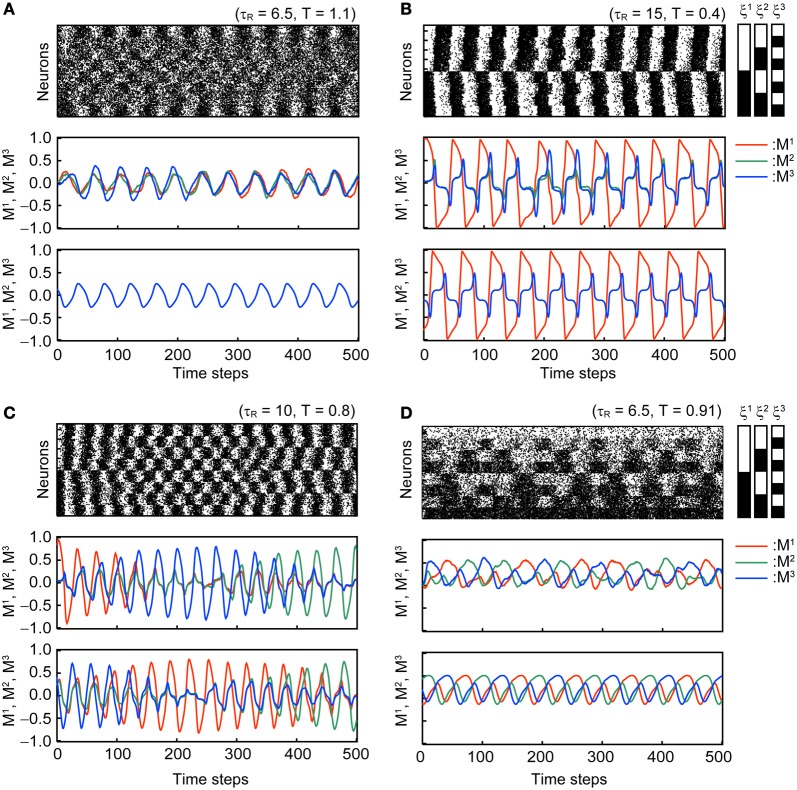
**Oscillatory states of the associative network with *U*_se_ = 0.1, *b* = 0.2, and τ_*F*_ = 2.** The first and second panels of each subfigure are the simulation results in the same format as in Figure 2. The third panel of each subfigure shows the time course of the overlaps in the macroscopic mean field model. Subfigures **(A–D)** display the different modes of oscillation. **(A)** Oscillatory state with MED = 1 (OS1). **(B)** Oscillatory state with 1 < MED ≤ 2 (OS2). **(C)** Oscillatory state showing the symmetric oscillatory pattern with 2 < MED ≤ 3 (OS3). **(D)** Oscillatory state showing the asymmetric oscillatory pattern with 2 < MED ≤ 3 (OS3).

Figure 3 shows typical time courses of the oscillatory states in the stochastic model with *N* = 10^4^ and the corresponding macroscopic mean field model. Dynamics of the mean field model is shown in the third panel in each subfigure and is consistent with that of the corresponding stochastic model.

In the OS1 state shown in Figure 3A, the network oscillates between the mixed state and the inverse of the mixed state; thus, the overlaps *M*^1^, *M*^2^, and *M*^3^ oscillate in phase and the *ED* = 1. The time course of overlaps in the macroscopic mean field model is shown in the third panel in Figure 3A.

In the OS2 state shown in Figure 3B, the network oscillates between one of the memory patterns and its inverse pattern; one of the overlap (*M*^1^ in the Figure 3B) oscillates with larger amplitude than others. The remaining two overlaps oscillate in phase. Because the model is symmetric, three possible patterns of oscillation exist and the realization of the oscillatory pattern depends on the initial state of the network.

In the OS3 state shown in Figures 3C,D, there are two submodes of oscillatory states. The first mode oscillates symmetrically between one of memory patterns and its inverted patterns, and appearance of the oscillation circulate among the three memory patterns (see Figure 3C ). The order of the three memory pattern randomly changes in the stochastic model. In the macroscopic mean field model, the oscillatory pattern with the orders *M*^1^ → *M*^2^ → *M*^3^ and *M*^3^ → *M*^2^ → *M*^1^ coexist (the oscillatory pattern with the order *M*^1^ → *M*^2^ → *M*^3^ is shown in the third panel of Figure 3C). The second mode shows asymmetric oscillation among three memory patterns (see Figure 3D) or among three inverted patterns. The order of circulation in the three memory (or inverted-memory) patterns is random in the simulation.

**Figure 4:**
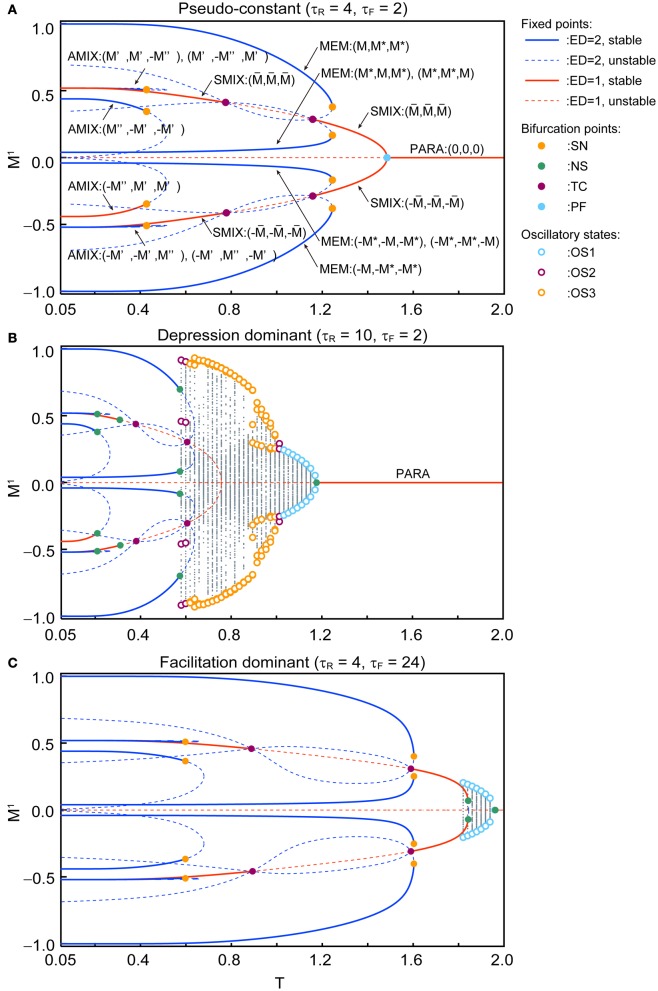
**Bifurcation diagrams with respect to *T* show changes in dynamical structure of the macroscopic mean field model with *U*_se_ = 0.1 and *b* = 0.2. (A)** In the pseudo-constant region the effects of dynamic synapses are relatively small (τ_*R*_ = 4 and τ_*F*_ = 2). Three overlaps (*M*^1^, *M*^2^, *M*^3^) on the steady state are represented by positive real numbers that satisfy *M* > M^*^ > 0, *M*′ > *M*″ > 0, $\bar{M}$ > 0. **(B)** The depression-dominant region (τ_*R*_ = 10 and τ_*F*_ = 2). **(C)** The facilitation-dominant region (τ_*R*_ = 4 and τ_*F*_ = 24). The red and blue curves indicate the fixed point where the ED is 1 and 2, respectively. The solid and dashed curves indicate stable and unstable fixed points, respectively. The orange, green, magenta, and cyan filled circles indicate the saddle-node (SN), Neimark–Sacker (NS), transcritical (TC), and pitchfork (PF) bifurcations, respectively. The cyan, magenta, and orange open circles indicate the maximum and minimum values of the oscillatory states OS1, OS2, and OS3, respectively. The gray dots indicate the orbit of the oscillatory state.

Figure 4 shows the qualitative difference in the bifurcation diagrams with respect to the noise intensity *T* in three different parameter regions: the pseudo-constant region (τ_*R*_ = 4 and τ_*F*_ = 2), the depression-dominant region (τ_*R*_ = 10 and τ_*F*_ = 2), and the facilitation-dominant region (τ_*R*_ = 4 and τ_*F*_ = 24). Here, we set *b* = 0.2 and *U*_se_ = 0.1.

In the pseudo-constant region (Figure 4A), the time constants τ_*R*_ and τ_*F*_ are relatively small, then the effect of the short-term plasticity quickly disappears, and the transmission efficacy of the dynamic synapses remains nearby its steady state. Figure 4A shows the bifurcation diagram with respect to the noise intensity *T* in the pseudo-constant region with τ_*R*_ = 4 and τ_*F*_ = 2. In the relatively low noise range with *T* < 0.4, AMIX, SMIX, and MEM states coexist as the stable fixed points. The absolute values of the overlaps decreased with *T*. As *T* increases, the fixed points that correspond to the AMIX states are destabilized via the saddle-node (SN) bifurcation at *T* = 0.429. Each of two SMIX states intersects with three unstable fixed points and becomes unstable at *T* = 0.781 via the transcritical (TC) bifurcation, which is stabilized again at *T* = 1.161 via another TC bifurcation. The two SMIX states disappear by coalescing with an unstable fixed point at *T* = 1.488 via the pitchfork (PF) bifurcation, and the stable fixed point that corresponds to the PARA state emerges. All six MEM states disappear at *T* = 1.248 via the SN bifurcation.

In the depression-dominant region (Figure 4B), τ_*R*_ is relatively large, and the effect of decreases in the releasable neurotransmitters remains long. In this region, the position of the fixed point shrink to the low-noise side and quasi-periodic circles that correspond to oscillatory states appear. As *T* increases, AMIX, SMIX, and MEM states are destabilized via the Neimark-Sacker (NS) bifurcations at *T* = 0.212, *T* = 0.311, and *T* = 0.576, respectively. The oscillatory states appear at *T* = 0.569 and exhibit quasi-periodic oscillation on an invariant circle. There exists a multi-stable state of the stable fixed point and quasi-periodic states on the range from *T* = 0.569 to *T* = 0.576. As *T* increases, OS2, OS3, and OS1 appear in this order. The oscillatory states disappear via the NS bifurcation at *T* = 1.180.

In the facilitation-dominant region (Figure 4C), τ_*F*_ is relatively large, and the effect of increase in the utilization parameter remains long. In this region, the range of the fixed points that correspond to the MEM, SMIX, and AMIX is expanded. The overall bifurcation structure is similar to that of the pseudo-constant region, but the SMIX state is destabilized at *T* = 1.845 via the NS bifurcation. Furthermore, the OS1 state appear at *T* = 1.811 and disappear at *T* = 1.964 via the NS bifurcation.

**Figure 5:**
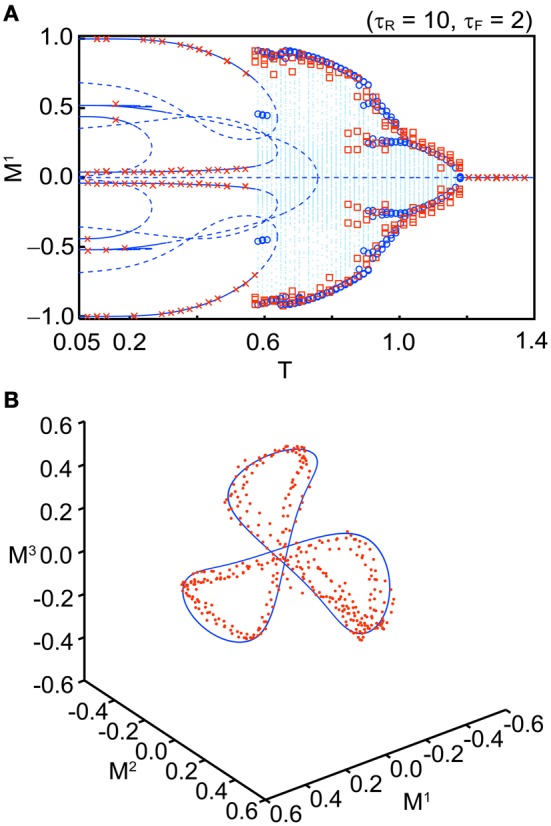
**Comparison between the stochastic model (*N* = 10^4^) and the macroscopic mean field model. (A)** A bifurcation diagram (*U*_se_ = 0.1, τ_*R*_ = 10, τ_*F*_ = 2, and *b* = 0.2). The blue curves, dots, and circles indicate the fixed points, orbits, and maximal or minimal values of the orbit in the macroscopic mean field model, respectively. The red crosses and squares indicate the corresponding simulation results. **(B)** Distribution of the orbits (*U*_se_ = 0.1, τ_*R*_ = 6.5, τ_*F*_ = 2, *T* = 0.91, and *b* = 0.2). The simulation result is indicated by red dots. The invariant circle in the macroscopic mean field model is indicated by the blue solid curve.

Figure 5A shows a bifurcation diagram for comparison between the macroscopic mean field model and the simulation when we set *U*_se_ = 0.1, τ_*R*_ = 10, τ_*F*_ = 2, *b* = 0.2, and *N* = 10^4^ with several initial values. The simulation shows good agreement with the corresponding macroscopic mean field model. Figure 5B shows an orbit of an OS3 state for *U*_se_ = 0.1, τ_*R*_ = 6.5, τ_*F*_ = 2, *b* = 0.2, and *T* = 0.91 in the simulation with *N* = 10^4^ (red dots) and in the macroscopic mean field model (the blue solid curve). The quasi-periodic orbit in the macroscopic mean field model also shows good agreement with the simulation. We have confirmed that the simulation result becomes closer to the macroscopic mean field model when *N* is increased.

**Figure 6:**
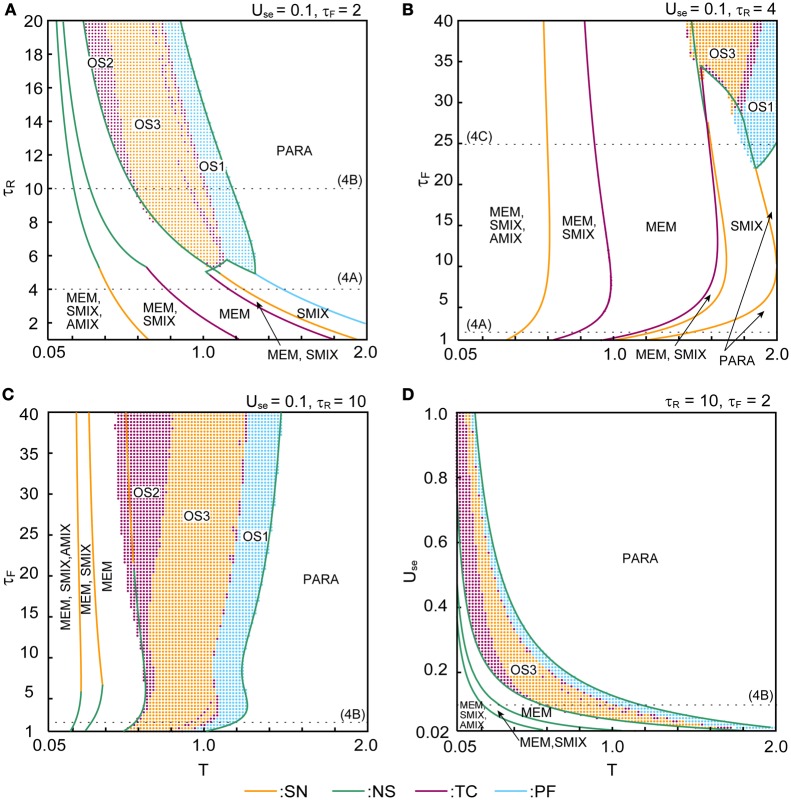
**Phase diagrams with respect to parameters that specify the properties of dynamic synapses. (A)** (τ_*R*_, *T*) phase diagram. **(B,C)** (τ_*F*_, *T*) phase diagrams. **(D)** (*U*_se_, *T*) phase diagram. The orange, green, magenta, and cyan curves indicate the sets of saddle-node (SN), Neimark–Sacker (NS), transcritical (TC), and pitchfork (PF) bifurcation points, respectively. The cyan, magenta, and orange dots indicate the oscillatory states OS1, OS2, and OS3, respectively. The dotted lines indicate the parameter points we used in Figure 4.

**Figure 7:**
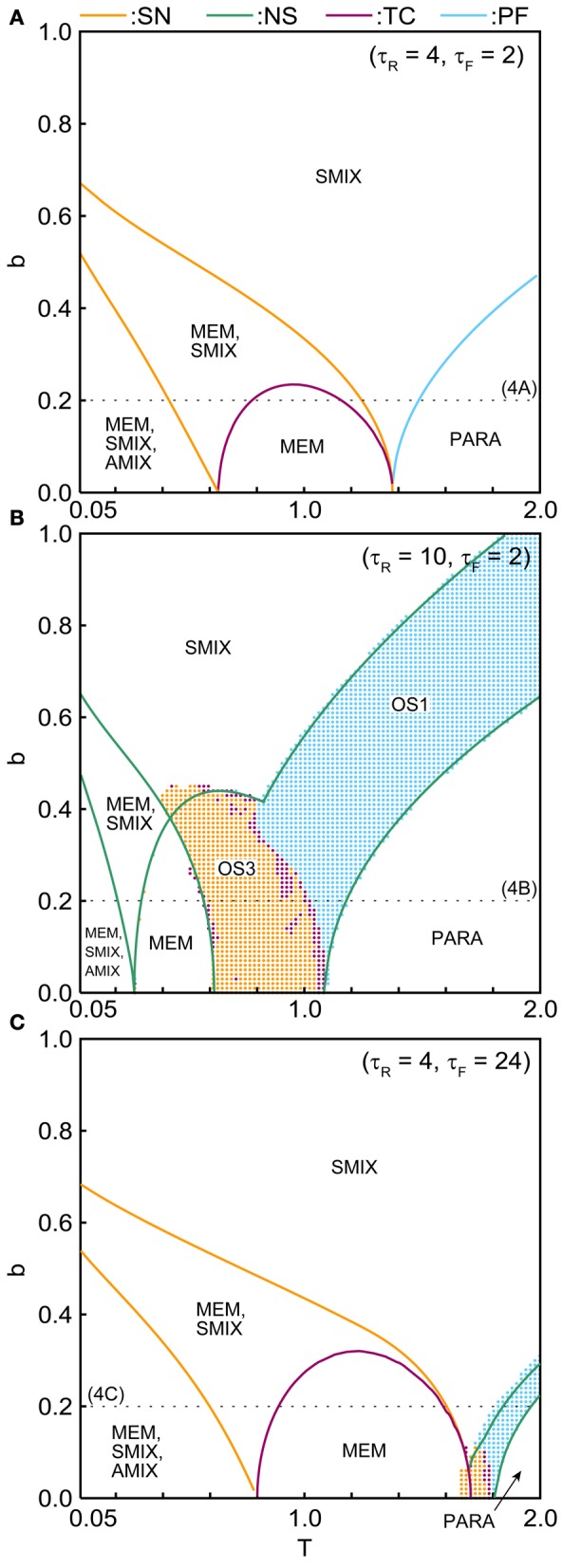
**(*b, T*) phase diagrams in (A) the pseudo-constant, (B) depression-dominant, and (C) facilitation-dominant ranges.** The format is the same as in Figure 6.

The phase diagrams in Figures 6, 7 show sets of bifurcation points that switch the stability of the fixed points and the distribution of the oscillatory states obtained by the brute-force methods. We calculated the time evolution of the macroscopic mean field model on each parameter points; the parameter points where the orbit converges to the oscillatory states are indicated by colored dots in Figures 6, 7. In the higher-noise boundary of the oscillatory state, the oscillatory states are separated by the supercritical type of the NS bifurcation; the region of the oscillatory states is well separated by the sets of the NS bifurcation. On the other hand, the oscillatory states appear with the subcritical type of NS bifurcation in the lower-noise boundary. Thus, the oscillatory states and the steady states coexist as multi-stable states in this region. Similar bifurcation structure is found in the uniformly connected network (Katori et al., 2012).

The (*T*, τ_*R*_) phase diagram in Figure 6A shows changes in the dynamical properties of the network from the pseudo-constant region to the depression-dominant region. As τ_*R*_ increases, the regions of the stable fixed point of MEM, SMIX, and AMIX shrink, while the regions of the PARA state and the oscillatory states expand. The (*T*, τ_*F*_) phase diagram shown in Figure 6B illustrates the dynamical properties from the pseudo-constant region to the facilitation-dominant region. As τ_*F*_ increase, the regions of MEM, SMIX, and AMIX expand, while the region of the PARA state shrinks. Furthermore, the oscillatory states appear. As τ_*F*_ increases from the depression-dominant region (Figure 6C), the regions of the oscillatory states expand. As *U*_se_ increases, the region of the PARA state expands, while regions of other states shrink.

The (*T, b*) phase diagrams in Figure 7 show that the dynamical properties of the network depend on the correlation level between the memory patterns. As *b* increases, the region of the SMIX state expands, while regions of the other states shrink. In the depression-dominant range (Figure 7B), as the correlation level *b* increases, the region of the OS3 state shrinks but that of OS1 state remain, which corresponds to the oscillatory state between SMIX states. In the facilitation-dominant range (Figure 7C), the overall bifurcation structure is similar to that of the pseudo-constant range, but the region of MEM states expands.

## 4. Discussion

In this study, we investigated the dynamical properties of an associative memory network composed of a stochastic neural network with both short-term depression and facilitation synapses on the basis of the macroscopic mean field model. We analyzed the behavior of the network in broad ranges of parameters that specify the noise intensity and the properties of the dynamic synapses. We found that the associative memory network exhibits the variety of dynamics, including the memory state, SMIX and AMIX, and several modes of the oscillatory states, and that its properties change with various types of bifurcations.

The performance of the memory retrieval can be characterized by the appearance of the MEM state in which the state of the network successfully converges to one of the memory patterns. In addition to the MEM state, in the relatively-low-noise range, there exists SMIX and AMIX states that correspond to pseudo-memory patterns. In this parameter range, the retrieval of the memory pattern is not assured and depends on the initial state of the network. In the high-noise range, the network tends to the PARA state, which corresponds to the state in which the pattern of neural activity is disrupted and randomized because of the noise. We classified the oscillatory states into three modes according to the ED. The OS1 state corresponds to oscillation between the pseudo-memory patterns, and it appears in the relatively high noise range. The OS2 state is the oscillation between one of the memory patterns and its inverse pattern, and it appears next to the MEM state. The OS3 state is the transitive state between memory patterns and their inverse patterns. Such transitive dynamics is related to the itinerant dynamics in terms of chaotic dynamics (Tsuda et al., 1987; Adachi and Aihara, 1997; Kanamaru et al., 2013).

The appearance of the above mentioned states of the network depends on the properties of the dynamic synapses (Figure 6) and on the correlation level between memory patterns (Figure 7). In the pseudo-constant region (Figure 4A), the state of the network converges to one of the fixed points like the conventional associative memory model (Anderson and Bower, 1972; Nakano, 1972; Hopfield, 1982). In the depression-dominant region, which is archived by increasing the recovery time constant τ_*R*_ from the pseudo-constant region, the area of successful memory retrieval shrinks, whereas the oscillatory states appear as shown in Figure 6A. Increase in the fraction of neurotransmitter-release *U*_se_ intensifies the influence of the depression. As *U*_se_ increase, the area of the PARA state expands, whereas the areas of other states shrink (Figure 6D). In the facilitation-dominant region, which is archived by increasing the time constant τ_*F*_ from the pseudo-constant region, the area of the memory retrieval expands (Figures 6B, 7C), which suggests that the facilitation synapses contribute to the memory retrieval (Mongillo et al., 2008). As the correlation level among memory patterns increases (Figure 7), the network loses the ability to retrieve the memory pattern, and the state of the network tends to become the pseudo-memory pattern. In the region of the oscillatory states, the oscillatory state among the memory patterns shrinks, whereas the oscillatory state between pseudo-memory patterns remains (Figure 7B).

These results have implications regarding brain functions. The distribution of facilitation and depression synapses in the brain varies according to the region of the brain. Many facilitation synapses exist in the prefrontal lobe and, whereas many depression synapses appear in the parietal lobe (Wang et al., 2006). The facilitation synapses may form a synaptic working memory and contribute to the prefrontal function, which requires a flexible executive function. Conversely, the depression synapses might be involved in memory search or mental rotation, which requires to imagine to handle an object in the parietal cortex (Tagaris et al., 1996). The oscillatory states OS3 observed in the present model correspond to the states that the neural network sequentially retrieves stored memory patterns. The oscillatory state appears with the incorporation of depression synapses. Furthermore, the area of the oscillatory state expands with increase in the time constant of the facilitation process. These findings imply that the depression and facilitation synapses contribute to various brain functions e.g., a generation of sequential actions or the flexible information representation (Katori et al., 2011).

The main findings of this work are consistent with previously reported studies on associative memory networks, and we revealed further details of the network dynamics. In the previous study on the associative memory network with both depression and facilitation synapses by Torres et al. (2007), the mean activities with active and inert neurons are considered to construct the mean field model, in which the number of the variables in the model is on the order of *p*. On the other hand, in our present study, we constructed the mean field model formulated with the sublattice method that enables to analyze the non-homogeneous network structure of the associative memory network; the number of the variables is on the order of 2^*p*^. In the case with *p* = 1, these two mean field models are equivalent, whereas these are differences in cases with *p* ≥ 2. Here we discussed the case with *p* = 3 and reported that the associative memory network exhibits a variety of dynamical states, including the memory and pseudo-memory states, as well as several oscillatory states among memory patterns. Furthermore, we reported the dependency of these states on the noise level and the parameters that specify the properties of the dynamic synapses, including details of bifurcation structure.

Although, we have considered the properties of the steady state and the oscillatory state as the attractors in the present study, properties of a transient process of memory retrieval should be evaluated. The relation between the stability of the memory retrieved states and irregularity of the neural activity (Mongillo et al., 2012) remains to be further investigated. In the present study, we used a simple neuron model, namely the discrete-time and binary neuron model. Meanwhile, the behavior observed in the present model should be qualitatively and/or quantitatively evaluated in more realistic neuron models e.g., integrate-and-fire or Hodgkin–Huxley model in the future.

## Conflict of interest statement

The authors declare that the research was conducted in the absence of any commercial or financial relationships that could be construed as a potential conflict of interest.
